# Association between Neighborhood Conditions and Mental Disorders among Children in the US: Evidence from the National Survey of Children's Health 2011/12

**DOI:** 10.1155/2018/5914315

**Published:** 2018-04-23

**Authors:** Sushma Dahal, Monica H. Swahn, Matthew J. Hayat

**Affiliations:** Division of Epidemiology and Biostatistics, School of Public Health, Georgia State University, Atlanta, GA, USA

## Abstract

**Background:**

This study examines the association between mental disorders and neighborhood conditions in a nationally representative sample of US children.

**Methods:**

Data from US children aged 6–17 years (*N* = 95,677) were obtained from the 2011/12 National Survey of Children's Health. Analysis examined neighborhood conditions and demographic and psychosocial characteristics including adverse childhood experiences (ACEs), parental mental health status, and the associations with any current diagnosed mental disorders (ACDMD). ACDMD was a composite variable derived from four childhood mental disorders examined. We computed descriptive statistics and logistic regression analyses.

**Results:**

Approximately 14% children had ACDMD. Of the neighborhood factors examined, nonsupportive neighborhood (AOR 1.37, 95% CI: 1.10, 1.71) was significantly associated with ACDMD in the multivariable models. Similarly, mother's mental health (AOR 1.84, 95% CI: 1.39, 2.43) and ACEs (e.g., AOR for 5–9 ACEs 6.36, 95% CI: 4.67, 8.65) were also found to be strongly associated with mental disorders.

**Conclusion:**

Our findings show that parental poor mental health, living in a nonsupportive neighborhood, and ACEs were important risk factors for child mental disorders. While more research is needed, children who have had early trauma and who reside with parents and caretakers with poor mental health are in need of additional services and treatment.

## 1. Introduction

Mental disorders among children in the U.S. are important public health concerns because of relatively high prevalence, early onset, and socioeconomic impacts [1]. According to previous surveillance reports, 13–20% of children below 18 years living in the U.S. have a mental disorder in a given year. Among children aged 3–17 years, the most common mental disorders are attention deficit hyperactivity disorder (ADHD), behavioral or conduct problems, anxiety disorder, depression, and autism spectrum disorder [2]. Risk factors for mental disorders among children and adolescent range from biological factors such as genetic susceptibility and head injury to social factors such as poverty and community disorganization [3]. Social factors include the circumstances in which people are born, grow, live, work, and age [4]. The neighborhood social and built environment are key components in child development and its impact on children is specific [5–7]. This is the case because of their rapid biological and psychological developmental process. Moreover, research shows that the effect of the neighborhood environment on children's mental health can be important even before age 10 [8].

Though there has been a rapid increase in the assessments of the built environment and neighborhood factors and their impact on health more broadly, particularly in the U.S., so far most of this research has focused on adult populations [9]. Similarly, available literature based on recent data on neighborhood conditions and mental health of US children is limited. Moreover, very few previous studies are based on nationally representative data [10]. The limited available literature suggests an association between common mental disorders among young people and neighborhood problems such as poor living conditions, economic deprivation, violence, and victimization [8, 11].

Improving our understanding of how the neighborhood related factors can shape the mental health of children has implication for both prevention and treatment of mental disorder among children as well as for planning programs at neighborhood level to potentially reduce the mental health burden. In this study, we examined the association between neighborhood conditions and common mental disorders among U.S. children aged 6–17 years and also specifically controlled this for a number of factors that may serve as confounders such as parent's mental health, parent's education, and adverse childhood experiences (ACEs) [9, 12–15]. Our key contribution to the literature is in providing new empirical findings with respect to community factors and their associations with each of the key mental disorders commonly reported among children (i.e., ADHD, depression, anxiety problem, and behavioral/conduct problem) in a more recent and nationally representative survey [16].

## 2. Methodology

Data for this study was obtained from the publicly available 2011/12 National Survey of Children's Health (NSCH), a national cross-sectional telephone survey containing information about 95,677 children aged 0–17 years [17]. The methodology for the NSCH involves a complex survey design conducted using a list-assisted random-digit-dial (RDD) sample of landline and RDD sample of cell-phone numbers [17]. Respondents to the survey were a parent or guardian who had knowledge about the health and health care of the sampled child and who provided the information about the child. More specific details of the NSCH methodology are provided elsewhere [16–18]. Our analyses were restricted to children aged 6–17 years because of our interest in specific mental disorders that would very rarely be diagnosed in a child under the age of 6 years.

We examined five outcome variables in our study. These are current diagnosed “ADHD,” “depression,” “anxiety problem,” “behavioral or conduct problem,” and a composite variable reflecting any or more of these four diagnoses and labeled as “any current diagnosed mental disorder (ACDMD)” which is also our primary outcome variable. The presence of the first four conditions was identified if the respondent affirmed a diagnosis by a medical doctor and also affirmed that the child currently had the condition (refer to Table 1). Similarly, the composite variable “ACDMD” was recorded as positive if the child currently had at least one of these four mental disorders. We selected these four disorders because of their high prevalence among children aged 3–17 [2]. Condition such as autism spectrum disorder was not included because of its early age of onset (<3 years) and genetic susceptibility [19].

The independent variables were five neighborhood conditions: (1) neighborhood amenities or resources, (2) neighborhood distracting element, (3) supportive neighborhood, (4) neighborhood safety, and (5) school safety. The survey questions used for each of these variables are listed in Table 1. Scoring for each of these five conditions was conducted as per NSCH guidelines. For example, we obtained the “*neighborhood amenities score*” by summing the “yes = 1” response to each of the 4 questions (Table 1). This score that ranged from 0 (no amenities) to 4 (all four amenities) was identified as “neighborhood amenities count” and it was calculated for only those households that gave a valid response to all the four questions. Likewise, neighborhood cohesion score was calculated for only those households that gave a valid response to at least three questions (out of total four questions as provided in Table 1). The score for questions on neighborhood cohesion ranged from 1 (strongly agree) to 4 (strongly disagree). Those households with the mean score 0 to less than 2.25 were categorized as a supportive neighborhood, and those with score 2.25 to 4 were categorized as not supportive [20].

To create the variable “number of adverse childhood experiences or ACEs,” we used nine questions listed in Table 1 that were based on the Behavioral and Risk Factor Surveillance System (BRFSS) ACE module and a review of life course stressors in children's lives by a Technical Expert Panel [20]. The household poverty level was classified as per federal poverty level (FPL) guideline. We also assessed father's and mother's mental health separately by question, “Would you say that, in general, (sample child's father's or mother's) mental and emotional health is excellent, very good, good, fair, or poor?”

We summarized the data using descriptive statistics and reported unweighted frequency and weighted percentage results. Sampling weights were applied to enable a nationally representative sampling, allowing for generalizability of the results to noninstitutionalized children aged 6–17 years. Data analyses were conducted using SAS version 9.4. Logistic regression analyses were used to examine the associations. Bivariate logistic regression models were fit with each independent variable and the primary outcome variable. Statistically significant variables were retained in the multivariable model. Association of variables in the final multivariable model was examined with all the five outcome variables separately.

## 3. Results

### 3.1. Descriptive Statistics

A total of 65,680 children aged 6–17 years were included in the analytic sample of whom 8,972 (13.66%) had any current diagnosed mental disorder (ACDMD). We found that among children aged 6–17 years, 9.97% had ADHD, 4.16% had anxiety problem, 3.73% had behavioral/conduct problem, and 2.76% had depression.  Table 2 summarizes the sociodemographic characteristics of the respondents. The prevalence of current diagnosed mental disorder increased with age from about 9% among 6–8 year olds to 16% among 15–17 year olds. Mental disorders were higher among males (17%), non-Hispanic Whites (16%), in families with income less than 100% FPL (17%), and among children with a father and/or a mother with poor mental health (23% and 26%, resp.).

We also examined associations between ACEs and mental disorders. The prevalence of mental disorders increased almost five times among children who experienced 5–9 adverse events (39%) compared to those without any ACEs (8%). Comparing across all variables, children with 5–9 adverse experiences had the highest percentage of mental disorder (39.20%), followed by those whose mother had fair or poor mental health (26.23%) (Table 2).

Approximately 4% of children lived in neighborhood with no amenities and a similar percentage lived in neighborhood with all the three detracting elements. About 16% of the children lived in a neighborhood perceived to be “not supportive” by the parents. Among the children living in neighborhood with all three detracting elements, about 20% had mental disorder compared to 13% among children living in the neighborhood with no detracting elements. Similarly, about 18% of children living in not supportive neighborhood had mental disorder compared to 13% children living in supportive neighborhood (Table 3).

### 3.2. Bivariate and Multivariable Associations between Variables

In the bivariate logistic regression analysis, our dependent variable was the composite variable ACDMD and we found that poor neighborhood conditions were significantly associated with increased odds of current diagnosed mental disorder among children (Table 3). Similarly, older children, males, of non-Hispanic White race, living in poverty with less than 100% FPL, with fair/poor father's or mother's mental health, and with higher number of ACEs had significantly increased odds of reporting a mental disorder in the unadjusted model (Table 2, unadjusted OR).

In the first multivariable model, we estimated the adjusted odds ratio (AOR) after controlling for the variables that were significantly associated with mental disorder in the bivariate model. We found that not having supportive neighborhood (AOR 1.37 CI: 1.10, 1.71), being male (AOR 1.97 CI: 1.71, 2.27), age greater than 6–8 years, non-Hispanic White race, fair/poor mental health of father (AOR 1.35 CI: 1.01, 1.81), fair/poor mental health of mother (AOR 1.84 CI: 1.39, 2.43), and higher number of ACEs were significantly associated with mental disorder. The highest adjusted OR were among those children having 5–9 ACEs (AOR 6.36 CI: 4.67, 8.65), followed by those having 3-4 ACEs (AOR 3.01 CI: 2.30, 3.94), and those having 2 ACEs (AOR 2.26 CI: 1.77, 2.88) (Table 3, adjusted OR).

Factors that were significantly associated with the composite variable in the first multivariable model were supportive environment, child's age, child's sex, child's race, father's mental health, mother's mental health, and number of adverse childhood experiences. These factors were included in the second multivariable model and the association was examined for all our dependent variables: current diagnosed mental disorder, ADHD, depression, anxiety problems, and behavioral/conduct problems separately (Table 4). Children living in not supportive neighborhoods had significantly higher odds of current diagnosed mental disorder (AOR*ϯ* 1.32, CI: 1.08, 1.61), ADHD (AOR*ϯ* 1.33, CI: 1.06, 1.67), and anxiety problem (AOR*ϯ* 1.39, CI: 1.02, 1.89) compared to children living in supportive neighborhoods. We found that, for both current diagnosed mental disorder and each category of mental disorders, a child having 5–9 ACEs had the highest AOR*ϯ* compared to a child not having any ACEs. For behavioral/conduct problems and depression, the AOR*ϯ* were as high as 13 and 10, respectively. Mother's fair or poor mental health was associated with AOR of 2.39 (CI: 1.50, 3.81) for depression and 3.00 (CI: 1.98, 4.55) for anxiety problem. Males had significantly higher odds than girls for ADHD, behavioral/conduct problems, and any current diagnosed mental disorder. Similarly, being non-Hispanic White was significantly associated with greater odds of ADHD, depression, anxiety problem, and current diagnosed mental disorder (Table 4).

## 4. Discussion

In this study, we examined the associations between neighborhood conditions and common mental disorders among nationally representative sample of U.S. children aged 6–17 years. We found that a diagnosis of a mental disorder varied substantially by the demographic and psychosocial characteristics of the child. In particular, a mental disorder was more commonly reported for boys and children who were white and for those children who had parents with poor mental health and who had any adverse childhood experiences. In terms of neighborhood conditions, we found that living in a nonsupportive neighborhood was associated with a mental disorder, even in adjusted analyses. More specifically, the association between parent's perceived nonsupportive neighborhood and mental disorder remained significant even after controlling for family and child's characteristics [OR = 1.37, 95% CI: 1.10, 1.70].

Resources in neighborhood like parks, libraries, and community centers promote healthy development of children [21]. However, in our study, the neighborhood amenities count was not an important factor for a mental disorder compared to the other neighborhood factors. This was surprising given that previous research demonstrates an association between few neighborhood amenities and anxiety and depression [11].

For school-age children who may be more proximal with the school environment than their neighborhood, school environment could possibly mediate or explain the mechanism of neighborhood influence [21]. In our study, children who went to school and whose parents perceived them to be never safe were twice as likely to have a mental disorder compared to children who went to school perceived to be safe. However, this finding was no longer significant in adjusted models.

Besides the effect of neighborhood factors, we found a strong association between parental mental health and ACEs and mental disorders among children. The associations between ACEs and mental disorders were particularly strong and remained significant even in fully adjusted models. Moreover, children having 5–9 ACEs had about 6 times the odds of also having a mental disorder compared to children not having any ACEs. Interestingly, we observed a dose response relationship between number of ACEs and a mental health diagnosis. These findings of the strengths between parental mental disorder, ACEs, and child mental disorders have been reported in previous studies [14, 22, 23].

Children of parents who are mentally ill or who have the history of mental disorders are at increased risk of mental disorders [11, 12]. This increased risk may be associated with several factors such as decreased parent-child interaction because of ill mental health of parents, poor parenting skills, limited communication, risk of child abuse, and other adverse factors in these families including the genetic susceptibility towards mental illness of these children [24]. Children of mothers with a history of depression or depressive symptoms, for example, are also found to be susceptible to the effect of neighborhood stressors [9]. In line with this research, we found that father's and mother's poor mental health was associated with a current diagnosed mental disorder among children. While father's poor mental health was significantly associated with depression and anxiety problem, mother's poor mental health was significantly associated with all the mental disorders examined: ADHD, depression, anxiety problem, and behavioral and conduct problem.

In our study, Hispanics, non-Hispanics Black, and multiracial or other non-Hispanics children were found to be significantly less likely to have mental disorder compared to non-Hispanic Whites. This difference may be linked with ethnic group concentration or ethnic group solidarity that protects the adolescents from the effect of material deprivation in the neighborhood [25, 26]. Alternatively, this may be linked with structural factors linked to health disparities such as less access to mental health care and service use among Black and Hispanics compared to whites [27], high unmet mental health needs among Latino children [28], and more stigma related concerns about mental health care among women of ethnic minorities [29]. In this study, we are not able to determine the underlying reasons for these differences.

*Limitations*. This study has several important limitations that should be considered when interpreting the findings. First, our analyses were restricted to examine four childhood mental disorders and the composite variable of these four. As such, the prevalence of mental disorders reported in this study will be lower and should not also be generalized to other forms of mental disorders not examined. Second, another important limitation pertains to the study participant, the respondents who were the parents or guardians of the children for which the data were collected. The information collected is based solely on the parents and caretakers and were not corroborated with other information. We suspect that because of social desirability biases, our findings may still represent an undercount of the true prevalence of mental disorders among children as some parents may not be willing to disclose such information and/or may also not know if their child had a diagnosis. Third, while this study used a complex survey design to collect the data, the phone surveys may yet produce another bias in that some people may not be willing to participate in these lengthy interviews over the telephone and as such may exclude information from some population groups. Fourth, our findings show that parental poor mental health was a key risk factor for child mental disorder. However, this finding may be exacerbated if parents who themselves may have poor mental health are more likely to perceive their child to also be of poor mental health. It is possible that there is a reporting bias that may explain some of these results which should be an important question for future research. Fifth, while we examined a number of community factors, there are many potential risk factors and psychosocial characteristics which would have been important to include in a study of mental disorders. These may include residential instability, family conflict, community transitions, and relationship of child with parents and peers and have been found to be associated with mental disorder among children in previous research [3, 8, 30]. Sixth, another limitation is that we did not address the potential multilevel nature of data. Instead, we decided that applying the sampling weights were more essential to answering the research questions and to provide generalizable findings of our results since the data was collected to be nationally representative. As such we used aggregated neighborhood measures in a logistic regression framework at the individual level. Despite these limitations, this study provides estimates of the association between neighborhood conditions with the common mental disorders among children adjusting for variables like adverse childhood experiences in a large nationally representative sample.

Our findings have important public health implication as they underscore clear associations between some neighborhood conditions and poor mental health among U.S. children. Moreover, future research needs to examine more specifically the potential causal mechanism that links neighborhood disadvantage to poor mental health while also factoring ACEs and particularly mother's mental health. Additionally, it will be important to specify whether it is the social or physical and built environment that is the potential driver for poor mental health. In the short term, our findings suggest that children who live in disadvantaged neighborhoods will need additional support to not exacerbate other factors that may contribute to their poor mental health.

## Figures and Tables

**Table 1:** Survey questions for different conditions.

Condition	Survey questions
ADHD, depression, anxiety problem, behavioral or conduct problem	(a) Has a doctor or other health care provider ever told you that child had (the condition)? (b) Does the child currently have (the condition)?

Neighborhood amenities	Please tell me if the following places and things are available to children in your neighborhood, even if (child) does not actually use them (1) Sidewalks or walking paths? (2) A park or playground area? (3) A recreation center, community center, or boys' or girls' club? (4) A library or bookmobile?

Neighborhood detracting elements	In your neighborhood, (1) Is there litter or garbage on the street or sidewalk? (2) How about poorly kept or rundown housing? (3) How about vandalism such as broken windows or graffiti?

Supportive neighborhood	How much you agree or disagree with each of these statements about your neighborhood or community? (1) “People in this neighborhood help each other out.” (2) “We watch out for each other's children in this neighborhood.” (3) “There are people I can count on in this neighborhood.” (4) “If my child were outside playing and got hurt or scared, there are adults nearby who I trust to help my child.”

Neighborhood safety	How often do you feel the child is safe in your community or neighborhood? Would you say never, sometimes, usually, or always?

School safety	How often do you feel (he/she) is safe at school? Would you say never, sometimes, usually, or always?

Adverse childhood experience	(1) How often is it hard to get by on your family's income? (2) Did child ever see or hear any parents, guardians, or any other adults in his/her home slap, hit, kick, punch, or beat each other up? (3) Was child ever treated or judged unfairly because of race or ethnic group? (4) Was child ever the victim of violence or witnessed any violence in his/her neighborhood? Did child ever live with a parent or guardian: (5) Who got divorced or separated after the child was born? (6) With a parent or guardian who died? (7) With a parent or guardian who served time in jail or prison after the child was born? (8) With anyone who was mentally ill or suicidal, or severely depressed for more than a couple of weeks? (9) With anyone who had a problem with alcohol or drugs?

*Source*. CDC National Center for Health Statistics. SLAITS-National Survey of Children's Health, 2012 [16].

**Table 2:** Sociodemographic characteristics and any current diagnosed mental disorder (ACDMD) among children (total unweighted count = 65,680 total unweighted count of children with current diagnosed mental disorder = 8,927).

Variable	Total	ACDMD		ACDMD	ACDMD
	Unweighted count (weighted%)	Unweighted count (weighted%)		Unadjusted OR (95% CI)	Adjusted OR (95% CI)
		Yes	No		
*Child's age (years)*					
6–8	15420 (24.59)	1449 (9.40)	13909 (90.60)	Reference	Reference
9–11	15659 (24.77)	2239 (14.32)	13372 (85.68)	1.61 (1.38, 1.90)	1.47 (1.19, 1.81)
12–14	16157 (24.95)	2398 (14.64)	13675 (85.36)	1.65 (1.42, 1.92)	1.44 (1.17, 1.77)
15–17	18444 (25.69)	2847 (16.02)	15508 (83.98)	1.84 (1.59, 2.13)	1.57 (1.27, 1.93)

*Child's sex*					
Male	33986 (51.21)	5755 (17.02)	28051 (82.98)	1.83 (1.65, 2.04)	1.97 (1.71, 2.27)
Female	31607 (48.79)	3172 (10.06)	28333 (89.94)	Reference	Reference

*Child's race*					
Hispanic	8073 (22.32)	868 (9.55)	7173 (90.45)	0.55 (0.46, 0.66)	0.53 (0.39, 0.71)
White, non-Hispanic	43153 (53.72)	6259 (16.10)	36720 (83.90)	Reference	Reference
Black, non-Hispanic	6177 (14.22)	787 (13.35)	5361 (86.65)	0.80 (0.69, 0.93)	0.53 (0.40, 0.69)
Multiracial/other, non-Hispanic	6712 (9.74)	845 (11.09)	5832 (88.91)	0.65 (0.55, 0.76)	0.48 (0.39, 0.60)

*Poverty level of the household*					
<100% FPL	9135 (20.66)	1740 (16.91)	7342 (83.09)	1.59 (1.38, 1.83)	1.14 (0.89, 1.47)
100–199% FPL	11449 (21.42)	1814 (14.63)	9575 (85.37)	1.34 (1.16, 1.54)	0.96 (0.79, 1.18)
200–399% FPL	20329 (28.86)	2519 (12.80)	17732 (87.20)	1.15 (1.00, 1.32)	0.91 (0.77, 1.07)
400 or more FPL	24767 (29.06)	2860 (11.36)	21815 (88.64)	Reference	Reference

*Father's mental health*					
Excellent/very good/good	49441 (94.75)	5505 (11.19)	43759 (88.81)	Reference	Reference
Fair/poor	2359 (5.25)	529 (23.14)	1798 (76.86)	2.39 (1.94, 2.95)	1.35 (1.01, 1.81)

*Mother's mental health*					
Excellent/very good/good	56091 (91.79)	6688 (12.13)	49196 (87.87)	Reference	Reference
Fair/poor	3965 (8.21)	1139 (26.23)	2796 (73.77)	2.58 (2.19, 3.03)	1.84 (1.39, 2.43)

*Father's highest education*					
Less than high school	3882 (14.06)	460 (10.23)	3390 (89.77)	0.86 (0.69, 1.08)	0.77 (0.56, 1.07)
High school graduate	11353 (24.33)	1539 (13.34)	9772 (86.66)	1.16 (1.01, 1.34)	1.03 (0.86, 1.24)
More than high school	35852 (61.61)	3971 (11.70)	31751 (88.30)	Reference	Reference

*Mother's highest education*					
Less than high school	4364 (14.11)	590 (11.85)	3752 (88.15)	0.88 (0.73, 1.07)	0.80 (0.57, 1.13)
High school graduate	10992 (21.86)	1634 (14.78)	9315 (85.22)	1.14 (1.00, 1.29)	0.94 (0.77, 1.14)
More than high school	44026 (64.03)	5537 (13.21)	38325 (86.79)	Reference	Reference

*Number of ACEs*					
0	33718 (46.58)	2841 (8.12)	30760 (91.88)	Reference	Reference
1	15526 (25.89)	2085 (12.93)	13377 (87.07)	1.68 (1.47, 1.92)	1.73 (1.46, 2.05)
2	7026 (12.28)	1341 (17.76)	5638 (82.24)	2.44 (2.09, 2.86)	2.26 (1.77, 2.88)
3-4	6051 (10.86)	1571 (24.59)	4442 (75.41)	3.69 (3.14, 4.33)	3.01 (2.30, 3.94)
5–9	2558 (4.39)	1007 (39.20)	1537 (60.80)	7.29 (6.01, 8.86)	6.36 (4.67, 8.65)

FPL = federal poverty level; adjusted odds ratio (AOR) from multivariable model I adjusted for neighborhood amenities, detracting element count, supportive neighborhood, neighborhood safety, school safety, age, sex, race, poverty, father's mental health, mother's mental health, father's education, mother's education, and number of ACEs.

**Table 3:** Neighborhood characteristics and ACDMD among children (total unweighted count = 65,680 total unweighted count of children with current diagnosed mental disorder = 8,927).

Variable	Total	ACDMD		ACDMD	ACDMD
	Unweight count (weighted%)	Unweighted count (weighted%)		Unadjusted OR (95% CI)	Adjusted OR (95% CI)
		Yes	No		
*Neighborhood amenities count*					
4	33427 (53.52)	4317 (12.91)	28969 (87.09)	Reference	Reference
3	15747 (24.33)	2197 (13.97)	13490 (86.03)	1.10 (0.97, 1.24)	0.97 (0.84, 1.13)
2	8218 (12.14)	1216 (14.71)	6965 (85.29)	1.16 (1.00, 1.35)	1.05 (0.86, 1.29)
1	4107 (6.28)	637 (16.37)	3449 (83.63)	1.32 (1.08, 1.62)	1.06 (0.77, 1.46)
0	2385 (3.73)	366 (15.64)	2010 (84.36)	1.25 (0.93, 1.69)	1.22 (0.78, 1.91)

*Neighborhood detracting element count *					
0	47425 (71.99)	6151 (12.77)	41086 (87.23)	Reference	Reference
1	10973 (17.15)	1611 (15.26)	9314 (84.74)	1.23 (1.07, 1.41)	1.03 (0.86, 1.23)
2	4017 (7.11)	682 (15.82)	3308 (84.18)	1.28 (1.04, 1.58)	0.89 (0.64, 1.22)
3	2055 (3.75)	355 (20.15)	1689 (79.85)	1.72 (1.38, 2.20)	0.82 (0.50, 1.34)

*Supportive neighborhood*					
Yes	56248 (83.60)	7208 (12.89)	48827 (87.11)	Reference	Reference
No	7965 (16.40)	1566 (18.41)	6340 (81.59)	1.53 (1.32, 1.73)	1.37 (1.10, 1.71)

*Neighborhood safety*					
Always/usually	58708 (86.93)	7745 (13.29)	50721 (86.71)	Reference	Reference
Sometimes	4903 (11.03)	870 (16.30)	4006 (83.70)	1.27 (1.07, 1.51)	1.00 (0.73, 1.37)
Never	881 (2.04)	179 (18.60)	696 (81.40)	1.49 (1.10, 2.05)	1.04 (0.59, 1.85)

*School safety*					
Always/usually	59318 (92.64)	7757 (13.19)	51321 (86.81)	Reference	Reference
Sometimes	3198 (6.75)	702 (19.87)	2472 (80.13)	1.63 (1.35, 1.97)	1.33 (0.97, 1.83)
Never	261 (0.61)	71 (23.39)	189 (76.61)	2.01 (1.17, 3.45)	1.53 (0.62, 3.75)

Adjusted odds ratio (AOR) from multivariable model I adjusted for neighborhood amenities, detracting element count, supportive neighborhood, neighborhood safety, school safety, age, sex, race, poverty, father's mental health, mother's mental health, father's education, mother's education, and number of ACEs.

**Table 4:** Adjusted OR (AOR*ϯ*) for different categories of mental disorder.

Variables	ADHD	Depression	Anxiety problem	Behavioral/conduct problem	ACDMD
	AOR*ϯ* (95% CI)	AOR*ϯ* (95% CI)	AOR*ϯ* (95% CI)	AOR*ϯ* (95% CI)	AOR*ϯ* (96% CI)
*Child's age (year) *					
6–8	Reference	Reference	Reference	Reference	Reference
9–11	1.52 (1.20, 1.92)	2.90 (1.45, 5.81)	1.64 (1.21, 2.23)	1.13 (0.75, 1.71)	1.47 (1.20, 1.80)
12–14	1.52 (1.21, 1.90)	3.11 (1.66, 5.80)	1.61 (1.16, 2.24)	0.75 (0.50, 1.13)	1.47 (1.20, 1.79)
15–17	1.28 (1.02, 1.62)	6.40 (3.54, 11.55)	1.81 (1.33, 2.47)	0.95 (0.62, 1.46)	1.63 (1.33, 1.99)

*Child's sex*					
Male	2.41 (2.05, 2.83)	1.12 (0.81, 1.54)	1.08 (0.88, 1.33)	2.12 (1.52, 2.96)	1.90 (1.65, 2.18)
Female	Reference	Reference	Reference	Reference	Reference

*Child's race*					
Hispanic	0.41 (0.31, 0.56)	0.81 (0.48, 1.37)	0.38 (0.26, 0.56)	0.71 (0.43, 1.18)	0.46 (0.35, 0.59)
White, non-Hispanic	Reference	Reference	Reference	Reference	Reference
Black, non-Hispanic	0.57 (0.43, 0.75)	0.57 (0.32, 1.03)	0.35 (0.20, 0.60)	0.76 (0.51, 1.13)	0.53 (0.41, 0.69)
Multiracial/other, non-Hispanic	0.47 (0.37, 0.59)	0.48 (0.28, 0.84)	0.50 (0.36, 0.68)	0.69 (0.46, 1.01)	0.49 (0.40, 0.60)

*Supportive neighborhood*					
Yes	Reference	Reference	Reference	Reference	Reference
No	1.33 (1.06, 1.67)	1.07 (0.66, 1.73)	1.39 (1.02, 1.89)	1.17 (0.79, 1.72)	1.32 (1.08, 1.61)

*Father's mental health*					
Excellent/VG/good	Reference	Reference	Reference	Reference	Reference
Fair/poor	1.15 (0.84, 1.57)	1.71 (1.01, 2.88)	1.57 (1.05, 2.33)	1.43 (0.88, 2.33)	1.30 (0.98, 1.73)

*Mother's mental health*					
Excellent/VG/good	Reference	Reference	Reference	Reference	Reference
Fair/poor	1.43 (1.08, 1.89)	2.39 (1.50, 3.81)	3.00 (1.98, 4.55)	2.31 (1.46, 3.66)	1.84 (1.42, 2.40)

*Number of ACEs*					
0	Reference	Reference	Reference	Reference	Reference
1	1.69 (1.40, 2.02)	2.76 (1.81, 4.20)	1.94 (1.50, 2.52)	3.04 (2.07, 4.46)	1.73 (1.47, 2.03)
2	2.08 (1.62, 2.67)	3.95 (2.53, 6.16)	2.01 (1.49, 2.72)	4.87 (2.91, 8.17)	2.28 (1.81, 2.87)
3-4	2.79 (2.07, 3.75)	7.99 (4.75, 13.45)	2.31 (1.63, 3.27)	8.49 (5.36, 13.45)	2.98 (2.31, 3.84)
5–9	4.97 (3.48, 7.10)	10.31 (6.43, 16.54)	4.96 (3.45, 7.15)	12.53 (8.16, 19.24)	6.07 (4.45, 8.29)

Adjusted odds ratio (AOR*ϯ*) from the multivariable model II adjusted for supportive environment, child's age, child's sex, child's race, mother's mental health, father's mental health, and number.
